# A search for modifying genetic factors in *CHEK2*:c.1100delC breast cancer patients

**DOI:** 10.1038/s41598-021-93926-x

**Published:** 2021-07-20

**Authors:** Camilla Wendt, Taru A. Muranen, Lotta Mielikäinen, Jessada Thutkawkorapin, Carl Blomqvist, Xiang Jiao, Hans Ehrencrona, Emma Tham, Brita Arver, Beatrice Melin, Ekaterina Kuchinskaya, Marie Stenmark Askmalm, Ylva Paulsson-Karlsson, Zakaria Einbeigi, Anna von Wachenfeldt Väppling, Eija Kalso, Tiina Tasmuth, Anne Kallioniemi, Kristiina Aittomäki, Heli Nevanlinna, Åke Borg, Annika Lindblom

**Affiliations:** 1 Department of Clinical Science and Education, Karolinska Institutet, Södersjukhuset, Stockholm, Sweden; 2 Department of Obstetrics and Gynecology, Helsinki University Hospital, University of Helsinki, Helsinki, Finland; 3 Department of Molecular Medicine and Surgery, Karolinska Institutet, Solna, Stockholm, Sweden; 4 Department of Oncology, Helsinki University Hospital, University of Helsinki, Helsinki, Finland; 5 Department of Clinical Genetics and Pathology, Office for Medical Services, Region Skåne, Lund, Sweden; 6 Department of Oncology-Pathology, Karolinska Institutet, Solna, Stockholm, Sweden; 7 Department of Radiation Sciences, Oncology, Umeå University, Umeå, Sweden; 8 Department of Clinical Genetics, Department of Clinical Experimental Medicine, Linköping University, Linköping, Sweden; 9 Department of Immunology, Genetics and Pathology, Uppsala University, Uppsala, Sweden; 10 Department of Oncology, Sahlgrenska University Hospital, 41345 Göteborg, Sweden; 11 Department of Anaesthesiology, Intensive Care, and Pain Medicine, Helsinki University Hospital and University of Helsinki, Helsinki, Finland; 12 TAYS Cancer Centre and Faculty of Medicine and Health Technology, Tampere University; Fimlab Laboratories, Tampere University Hospital, Tampere, Finland; 13 Department of Medical and Clinical Genetics, University of Helsinki, Helsinki, Finland; 14 Department of Oncology and Pathology, Department of Clinical Sciences Lund, Lund University, Medicon Village, Lund, Sweden

**Keywords:** Cancer, Genetics, Risk factors

## Abstract

The risk of breast cancer associated with *CHEK2:*c.1100delC is 2–threefold but higher in carriers with a family history of breast cancer than without, suggesting that other genetic loci in combination with *CHEK2*:c.1100delC confer an increased risk in a polygenic model. Part of the excess familial risk has been associated with common low-penetrance variants. This study aimed to identify genetic loci that modify *CHEK2*:c.1100delC-associated breast cancer risk by searching for candidate risk alleles that are overrepresented in *CHEK2*:c.1100delC carriers with breast cancer compared with controls. We performed whole-exome sequencing in 28 breast cancer cases with germline *CHEK2*:c.1100delC, 28 familial breast cancer cases and 70 controls. Candidate alleles were selected for validation in larger cohorts. One recessive synonymous variant, rs16897117, was suggested, but no overrepresentation of homozygous *CHEK2*:c.1100delC carriers was found in the following validation. Furthermore, 11 non-synonymous candidate alleles were suggested for further testing, but no significant difference in allele frequency could be detected in the validation in *CHEK2*:c.1100delC cases compared with familial breast cancer, sporadic breast cancer and controls. With this method, we found no support for a *CHEK2*:c.1100delC-specific genetic modifier. Further studies of *CHEK2*:c.1100delC genetic modifiers are warranted to improve risk assessment in clinical practice.

## Introduction

Breast cancer aggregates in families and has a considerable inherited component. Approximately 20% of the genetic risk for breast cancer is explained by pathogenic mutations in the high-penetrance genes *BRCA1*, *BRCA2*, *TP53*, *STK11* and *PTEN*^1^. Other rare, intermediate-risk variants, such as *PALB2*, *CHEK2* and *ATM* account for about 5% of the inherited risk^2,3^ and common low-risk variants for another 18–19%^4–6^.

Checkpoint kinase 2 is a protein product of the *CHEK2* gene that localizes to chromosome 22q12.1. It is part of the network that responds to DNA damage in order to maintain genomic integrity^7^. The protein-truncating variant *CHEK2*:c.1100delC is associated with a two-threefold risk of breast cancer^8,9^. In women with familial aggregation of breast cancer, the risk is even higher. An odds ratio of up to 4.8 has been seen in women with a family history of breast cancer, which is equivalent to a 37% cumulative risk of breast cancer by the age of 70 years^8–10^. In addition, the c.1100delC allele has been associated with younger age at onset, a threefold increased risk of a second breast cancer, as well as a worse prognosis among women with oestrogen receptor-positive cancer^9,11,12^.

The considerably higher risk in women with a family history of breast cancer is in accordance with the suggested polygenic model where several susceptibility loci together confer a multiplicative effect on breast cancer risk^13,14^. The fact that the model also can be applied to *CHEK2*:c.1100delC carriers is supported by a study of low-risk breast cancer variants in 34 000 women with and without a family history of breast cancer. A polygenic risk score (PRS) that was based on the combined risk of 74 low risk variants was calculated. The result suggested that the polygenic risk score could be used to stratify risk in c.1100delC carriers and that the low-risk variants explained a part of the familial risk. The authors estimated that 20% of *CHEK2*:c.1100delC carriers with the highest PRS had an estimated lifetime breast cancer risk of > 30%. Correspondingly, 20% of carriers with the lowest PRS had an estimated lifetime risk of 14% which is close to the average population risk^15^. A synergistic effect between low-risk variants and *BRCA1* and *BRCA2* mutations has also been shown^16^. The risk for mutation carriers being affected is thus modified by other genetic variants and family history in addition to lifestyle factors. A risk prediction model, the Breast and Ovarian Analysis of Disease Incidence and Carrier Estimation Algorithm (BOADICEA) has been developed to calculate the lifetime risk of breast cancer, including carriers of a moderate-penetrance allele such as *CHEK2*:c.1100delC. The BOADICEA model allows risk stratification for established genetic and non-genetic risk factors^17^. Still, other causative gene variants possibly remain to be identified, since the previously identified low-, intermediate-, and high-risk genes cover less than half of the estimated heritable component. Characterising factors that increase the risk in carriers of moderate-risk alleles is important, in order to identify the high-risk group that benefits most from preventive interventions. In this study, we used whole-exome sequencing of a *CHEK2*:c.1100delC positive cohort with familial breast cancer, to identify putative risk modifying alleles. In the first phase we aimed to find candidate risk alleles for further validation in the second phase with larger cohorts of *CHEK2*:c.1100delC positive cases and controls.

## Results

**Figure 1:**
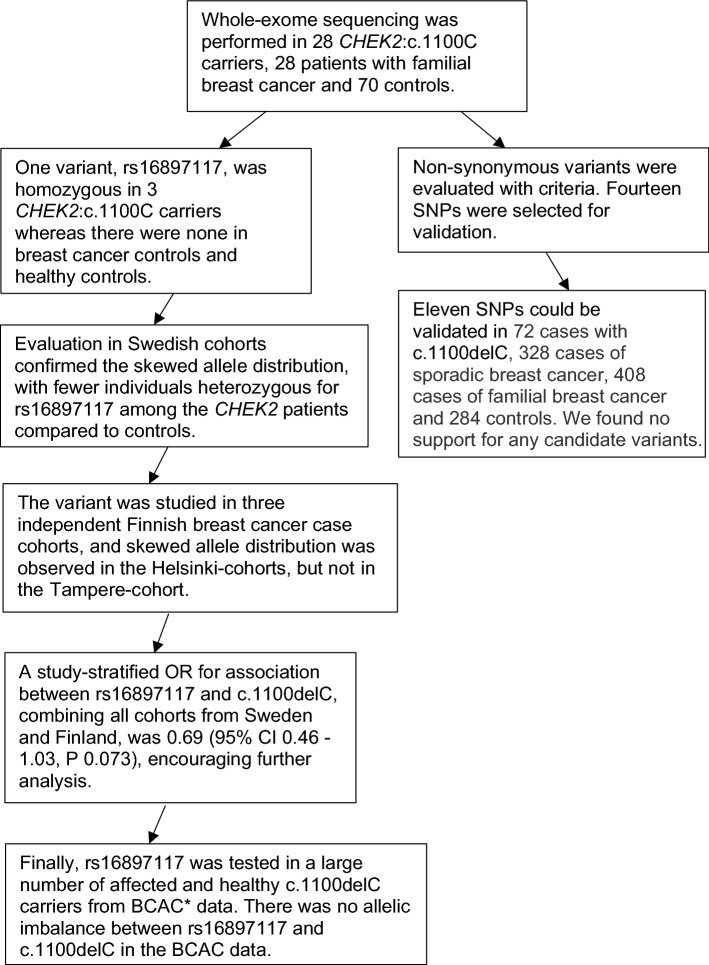
Flowchart describing the working process of evaluating genotype data in search of variants that specifically modify breast cancer risk in *CHEK2*:c.1100del carriers. *Breast Cancer Association Consortium.

We performed whole-exome sequencing in 28 breast cancer cases with germline *CHEK2*:c.1100delC, 28 familial breast cancer cases and 70 controls. Candidate alleles were selected for validation in larger cohorts (Fig. 1).

### Recessive variants

We analysed the exome sequencing data for a discovery of rare homozygous variants in *CHEK2*:c.1100C carriers, to identify risk alleles with recessive inheritance pattern. Only one variant was suggested, rs16897117. Among the 28 *CHEK2* carriers, there were 3 patients homozygous for rs16897117, whereas among the non-carrier breast cancer cases or healthy controls, there were no rs16897117 homozygotes. We set up to test the hypothesis of rs1689711 being a *CHEK2*:c.1100C risk modifier in larger sample collections, starting with 67 *CHEK2* patients, as well as 688 non-carrier breast cancer cases and 246 healthy controls. This study confirmed the skewed allele distribution, with fewer individuals heterozygous for rs16897117 among the *CHEK2* patients than among non-carrier patients or healthy controls. In a case-only analysis, the odds ratio between rs16897117 rare allele (A) and *CHEK2*:c.1100delC was 0.46 (95% confidence interval CI 0.17–1.04, *P* 0.053 (Table 1: SWEA1).

**Table 1:** Rs16897117 association with *CHEK2*:c.1100delC in a case-only analysis.

Cohort	rs16897117 in non-carriers GG–GA–AA	rs16897117 in c.1100delC carriers GG–GA–AA	OR [95% CI]	*P*
SWEA1	549–138–1	60–5–2	0.46 [0.17–1.04]	0.053
	(80%–20%–0.1%)	(90%–7%–3%)		
SWEA2	73–14–0	34–11–0	1.68 [0.62–4.47]	0.25
	(84%–16%–0%)	(76%–24%–0%)		
Helsinki1—unselected	1432–232–9	44–4–0	0.54 [0.14–1.50]	0.30
	(86%–14%–0.5%)	(92%–8%–0%)		
Helsinki1—additional familial	603–99–3	45–4–1	0.66 [0.20–1.71]	0.53
	(86%–14%–0.4%)	(90%–8%–2%)		
Helsinki2	841–119–5	26–2–0	0.52 [0.06–2.13]	0.57
	(87%–12%–0.5%)	(93%–7%–0%)		
Tampere	564–87–1	12–2–0	1.10 [0.11–4.92]	1.00
	(87%–13%–0.2%)	(86%–14%–0%)		
Combined			0.69 [0.46–1.03]	0.073

Next, we did another follow-up using 45 *CHEK2* carriers plus 87 familial breast cancer patients and 47 controls from the Swedish cohorts. None of the *CHEK2* carriers or the familial breast cancer patients were found to be homozygous for the rs16897117 variant. The only two homozygous individuals of this follow-up were identified in the control group. No skewness in allele distribution was observed in any of these groups (Table 1: SWEA2). The results seemed less clear, but to resolve this, we tested the association between rs16897117 and c.1100delC in a Finnish population, where the c.1100delC allele has a relatively high, 1.2%, frequency^18^. Genotyping of three independent patient series identified a single c.1100delC carrier patient, who was homozygous for rs16897117. The skewed allele distribution for rs16897117 was observed in the Helsinki cohorts, but not in the Tampere cohort. A study-stratified OR for association between rs16897117 and c.1100delC, combining all cohorts from Sweden and Finland, was 0.69 (95% CI 0.46–1.03, *P* 0.073), encouraging further analysis.

Finally, the genotype data for rs16897117 and c.1100delC were obtained from the OncoArray project of the Breast Cancer Association Consortium^5^. The availability of a good number of healthy c.1100delC carriers in the consortium data enabled a proper interaction analysis for c.1100delC, rs16897117, and breast cancer risk. In the BCAC data, there was no allelic imbalance between rs16897117 and c.1100delC (Table 2). A likelihood-ratio test comparing a breast cancer risk model with c.1100delC-rs16897117 interaction term with a plain model with c.1100delC and rs16897117 as independent risk factors did not support rs16897117 as a dosage-dependent risk modifier for c.1100delC carriers (Table 3). The BCAC data included four c.1100delC carriers, who were homozygous for rs16897117. These were all breast cancer cases, but the sample counts were too low for a reliable analysis.

**Table 2:** BCAC breast cancer cases and healthy controls with available data on *CHEK2*:c.1100delC and rs16897117 from the OncoArray project.

	rs16897117 in non-carriers GG–GA–AA	rs16897117 in c.1100delC carriers GG–GA–AA
Breast cancer cases	11,220–2247–111	147–38–4
	(83%–17%–1%)	(78%–20%–2%)
Healthy controls	17,561–3564–180	124–27–0
	(82%–17%–1%)	(82%–18%–0%)

**Table 3:** The breast cancer risk associated with *CHEK2*:c.1100delC and rs16897117 in the BCAC data.

	Plain model (OR)	Interaction model (OR)	P.LR
rs16897117	1.00 [0.95–1.06]	1.00 [0.94–1.05]	0.26
c.1100delC	2.03 [1.63–2.54]	1.92 [1.50–2.45]	
Interaction term		1.34 [0.80–2.28]	

The models were adjusted for BCAC study and 10 principal components.

### Coding non-synonymous candidate variants

In the discovery phase, exome sequencing data were analysed with a set of criteria in search of *CHEK2*:c.1100delC candidate variants. Fourteen non-synonymous variants were subject for testing, but only 11 were analysed due to technical issues with TaqMan probes (Table 4). The 11 missense variants detected in the *CHEK2*:c.1100delC carriers were evaluated in the validation phase. None of the variants could be replicated with similar patterns as in the discovery phase (Table 5). Thus, none was suggested to be a modifier of breast cancer risk in *CHEK2*:c.1100delC carriers.

**Table 4:** Variants selected in the discovery phase for further validation.

Chr	Variant type	SNP	Gene	1000g2014 oct eur	249 Swedes	200 Danes	ExAC NFE	CHEK2/CRC	CHEK2/FBC	CRC MAF	FBC MAF	CHEK2 MAF
1	SNV	rs2297809	CYP4B1	0.1302	0.1456	0.12	0.1457	2.07	1.65	0.1207	0.1518	0.25
1	SNV	rs4926600	CYP4A22	0.0805	0.0984	–	0.0868	3.06	1.29	0.0641	0.1518	0.1964
2	SNV	rs17860405	CASP10	0.0417	0.0382	0.0225	0.0409	7.51	1.80	0.0214	0.0893	0.1607
3	SNV	rs34492126	DLG1	0.0577	0.0542	0.0575	0.0535	2.35	1.64	0.0684	0.0982	0.1607
5	SNV	rs2287749	ADAM19	0.1163	0.1426	0.14	0.1350	1.39	1.53	0.1667	0.1518	0.2321
6	SNV	rs811925	PRDM1	0.2048	0.1767	0.1475	0.1758	2.04	1.17	0.1838	0.3214	0.375
9	SNV	rs34523498	CDK5RAP2	0.0328	0.0361	0.02	0.0295	4.18	1.33	0.0256	0.0804	0.1071
9	SNV	rs41305617	NOL8	0.0338	0.0221	–	0.0302	4.89	1.71	0.0219	0.0625	0.1071
11	SNV	rs8176786	NELL1	0.0547	0.0582	0.06	0.0529	2.44	1.55	0.0513	0.0804	0.125
11	SNV	rs117739035	SIGIRR	0.0358	0.0482	0.045	0.0347	4.18	1.55	0.0299	0.0804	0.125
12	SNV	rs7962217	VWF	0.0507	0.0542	0.0625	0.0545	2.09	1.71	0.513	0.0625	0.1071
15	SNV	rs35932273	LTK	0.0268	0.0321	0.01	0.0285	4.18	1.50	0.0256	0.0714	0.1071
16	SNV	rs152451	PALB2	0.0934	0.0683	0.0575	0.0955	5.53	0.93	0.0226	0.1339	0.125
20	SNV	rs34983477	TP53RK	0.0398	0.0502	0.0275	0.0473	2.09	1.20	0.0513	0.0893	0.1071

SNV, single nucleotide variant; SNP, single nucleotide variant; MAF, minor allele frequency; FBC, familial breast cancer cohort; CRC, cohort of healthy spouses in colorectal cancer families. Column 5–8 display MAF for the reference databases described in methods. CHEK2 cohort of *CHEK2*:c.1100delC carriers.

**Table 5:** Odds ratios for the 11 validated candidate alleles in *CHEK2*:c.1100delC familial breast cancer and sporadic breast cancer.

Gene/rs number	Cohort	Heterozygous	Homozygous	Wild type	Samples	Allele frequency	Odds ratio CI 95%	*P* value
PALB2 rs152451	CHEK2	6	0	64	70	0.043	0.623 [0.256–1.518]	0.293
	Familial	48	7	327	383	0.081	1.228 [0.792–1.904]	0.357
	Sporadic	31	4	262	297	0.066	0.977 [0.605–1.579]	0.925
	Controls	27	3	216	246	0.067	1	
PRDM1 rs811925	CHEK2	26	1	44	71	0.197	1.087 [0.679–1.739]	0.728
	Familial	129	18	243	390	0.211	1.187 [0.895–1.574]	0.232
	Sporadic	103	12	200	315	0.201	1.117 [0.831–1.503]	0.463
	Controls	76	9	170	255	0.184	1	
ADAM19 rs2287749	CHEK2	18	2	50	70	0.157	1.126 [0.671–1.891]	0.653
	Familial	103	7	256	366	0.160	1.149 [0.837–1.578]	1.149
	Sporadic	71	3	230	304	0.127	0.876 [0.621–1.236]	0.451
	Controls	59	7	191	257	0.142	1	
CYP4B1 rs2297809	CHEK2	16	3	47	65	0.167	1.005 [0.600–1.681]	0.985
	Familial	81	14	255	350	0.156	0.927 [0.678–1.266]	0.632
	Sporadic	45	17	227	289	0.137	0.795 [0.569–1.111]	0.179
	Controls	63	10	177	250	0.166	1	
VWF rs7962217	CHEK2	6	0	64	70	0.043	0.640 [0.261–1.566]	0.325
	Familial	38	3	340	381	0.058	0.876 [0.545–1.408]	0.583
	Sporadic	21	3	279	303	0.044	0.666 [0.392–1.133]	0.131
	Controls	25	3	209	237	0.065	1	
CASP10 rs17860405	CHEK2	8	1	60	69	0.072	1.078 [0.520–2.236]	0.840
	Familial	25	0	342	367	0.034	0.487 [0.288–0.823]	0.006
	Sporadic	19	0	270	289	0.033	0.469 [0.265–0.831]	0.008
	Controls	27	4	228	259	0.067	1	
DLG1 rs34492126	CHEK2	8	0	63	71	0.056	1.155 [0.505–2.642]	0.732
	Familial	41	2	344	387	0.058	1.194 [0.713–2.001]	0.499
	Sporadic	34	0	266	300	0.057	1.162 [0.675–2.001]	0.587
	Controls	19	2	213	234	0.049	1	
CDK5RAP2 rs34523498	CHEK2	5	0	65	71	0.036	1.125 [0.405–3.126]	0.791
	Familial	24	0	358	382	0.031	0.985 [0.518–1.874]	0.963
	Sporadic	22	1	285	309	0.039	1.231 [0.647–2.344]	0.526
	Controls	14	1	236	251	0.032	1	
TP53RK rs34983477	CHEK2	10	0	60	70	0.071	1.133 0.538–2.386]	0.743
	Familial	33	2	327	362	0.051	0.793 [0.481–1.309]	0.363
	Sporadic	30	2	255	287	0.059	0.927 [0.556–1.546]	0.927
	Controls	19	5	204	228	0.063	1	
SIGIRR rs117739035	CHEK2	5	0	65	70	0.0357	0.784 [0.293–2.102]	0.628
	Familial	45	0	349	394	0.057	1.282 [0.766–2.147]	0.342
	Sporadic	28	0	288	316	0.044	0.982 [0.558–1.726]	0.948
	Controls	23	0	232	255	0.045	1	
NELL1 rs8176786	CHEK2	3	1	46	53	0.031	0.392 [0.136–1.131]	0.073
	Familial	36	2	348	386	0.052	0.546 [0.340–0.879]	0.011
	Sporadic	33	2	264	297	0.055	0.588 [0.358–0.967]	0.035
	Controls	24	5	158	187	0.091	1	

CI, confidence interval.

## Discussion

We aimed to identify candidate risk variants that specifically modify risk in *CHEK2*:c.1100delC carriers through whole-exome sequencing of a small number of samples followed by validation in a case–control association study. No *CHEK2*:c.del1100C-specific candidate variants could be identified. Previously identified variants that modify breast cancer risk in *CHEK2*:c.1100delC carriers are also risk variants in the general breast cancer population. The common low-risk variants that predispose to breast cancer have also shown synergistic effects with *CHEK2*^14^. To our knowledge, no other genetic modifiers of *CHEK2*:c.1100delC have been suggested. Previously identified common alleles, associated with breast cancer in the general population have also been shown to modify risk in *BRCA1* and *BRCA2* mutation carriers, in a subtype specific manner^16^. A recent GWAS identified several novel loci that were associated with at least one tumour feature (ER-status, progesterone receptor status, tumour grade, human epidermal growth factor 2 receptor) and also loci that differed by the molecular subtype, luminal or non-luminal, of breast cancer^19^. The observations imply that tumour features should be taken into account when searching for candidate variants in *CHEK2:*c.del1100C carriers. Several loci that specifically modify risk in *BRCA1* and *BRCA2* carriers have also been found^16,20–29^. These are all low-risk susceptibility alleles identified through testing of candidates from breast cancer genome-wide association studies in *BRCA1/2* mutation carriers and through fine-mapping of candidate regions.

Future studies of *CHEK2*:c.1100delC modifying candidates could be done with more loose criteria in the discovery phase to increase the probability of finding good candidates for further testing. In accordance with previous findings, gene-specific modifiers are likely to be common low-risk variants. *CHEK2*:c.1100delC-specific modifiers may then rather be identified through large-scale genome-wide association studies. With this method, we found no support for a *CHEK2*:c.1100delC-specific genetic modifier. More studies of *CHEK2*:c.1100delC genetic modifiers are therefore warranted to improve risk assessment in clinical practice.

## Methods

In order to identify candidate variants, we conducted a discovery phase, where whole-exome sequencing was performed in 28 *CHEK2*:c.1100delC carriers with familial breast cancer, another 28 familial breast cancer patients and 70 healthy controls (spouses of colorectal cancer patients) from the Swedish cohorts. Candidate variants were validated in larger cohorts (Fig. 1).

### Sample preparation, discovery phase

Genomic DNA was subjected to whole-exome sequencing at the National Genomics Infrastructure in Uppsala, Sweden. Exome-enriched sequencing libraries were prepared using the Agilent SureSelectXT Human All Exon V5 XT2 + UTR kit (Agilent, Santa Clara, California, US). Cluster generation and 125 cycle paired-end sequencing was performed using the Illumina HiSeq 2500 system and v4 sequencing chemistry (Illumina, San Diego, California, US). Next-generation sequencing was performed at SciFiLab, University of Uppsala.

### Selection of non-synonymous candidate variants

After exome sequencing, all detected coding non-synonymous variants in the *CHEK2*:c.1100delC carriers were evaluated. The cases of hereditary breast cancer and the healthy controls (spouses of cases with hereditary colon cancer) served as genotyping controls in the work of identifying candidate alleles. Only variants passing a set of criteria, described below, were selected for further evaluation. The criteria were as follows:

### Allele frequency

Ratios of the allele frequencies of the variants were calculated. A ratio of 2.0 or more between *CHEK2*:c.1100delC cases and healthy controls and/or a ratio of 1.5 or more between *CHEK2*:c.1100delC cases and familial breast cancer cases was required.

### Gene function

Genes/variants that were selected should display a function of a putative cancer driver gene when evaluated by online genome browser databases (OMIM, GeneCards) and scientific publications available on PubMed.

### Reference databases

A more than 30% higher allele frequency in *CHEK2*:c.1100delC carriers compared with regional reference databases was required (ExAC non-Finnish population, 1000genome2014oct European, SweGen Variant Frequency Browser, exome sequencing data from 200 Danes^30^ and anonymous exome data from a cohort of 249 controls from the Department of Clinical Genetics, Karolinska University Hospital).

### Sequencing accuracy

Only variants with a sequencing accuracy of 65%, or more, in all study groups were included. The variants passing the selection criteria were functionally annotated using the in silico tools SIFT, Polyphen2 HDIV/HVAR, LRT, MutationTaster, FATHMM, RadialSVM, LR, and MutationAssessor.

### Validation of non-synonymous candidate variants

Eleven SNPs (rs2297809, rs17860405, rs8176786, rs34523498, rs117739035, rs34983477, rs152451, rs811925, rs7962217, rs34492126 and rs2287749) were genotyped using TaqMan SNP genotyping assay (Thermo Fisher Scientific, Waltham, Massachusetts, USA). rs35932273 was genotyped by Sanger sequencing following PCR. The candidates were validated in 72 cases with *CHEK2*:c.1100delC, 328 cases of sporadic breast cancer, 408 cases of familial breast cancer and 284 controls from the Swedish cohorts.

### Genotyping of a recessive candidate allele

Exome sequencing data were analysed in search of recessive candidate variants in *CHEK2*:c.1100delC carriers. One recessive variant, rs16897117, was suggested, as among the 28 *CHEK2* carriers, there were 3 patients homozygous for rs16897117, whereas among the non-carrier breast cancer cases or healthy controls, there were no rs16897117 homozygotes. The rs16897117 was further evaluated in Swedish and Finnish cohorts and in data from the Breast Cancer Association Consortium, BCAC.

### Swedish cohorts

The 28 samples from *CHEK2*:c.1100delC carriers analysed in the discovery phase were collected from the Department of Clinical Genetics, Karolinska University Hospital. A total of 112 samples from *CHEK2*:c.1100delC carriers were collected from the SWEA-study, a national Swedish collaboration aiming to study the prevalence of established breast cancer genes as well as to validate candidate genes and single nucleotide polymorphisms (SNPs) in Swedish women with familial breast- and ovarian cancer (72 and 112 samples for validation of non-synonymous variants and the recessive variant respectively). All *CHEK2*:c.1100delC carriers were previously affected by breast cancer except for two carriers who had been diagnosed with ovarian cancer. All cases of hereditary breast cancer were collected from the Department of Clinical Genetics, Karolinska University Hospital and had previously received counselling and screened negative for relevant high-risk genes (28 samples for discovery phase, 87 and 408 samples for validation of non-synonymous variants and the recessive variant respectively). Cancer-free spouses of colorectal cancer patients served as controls (70 samples for the discovery phase, 284 and 293 samples for validation). They were recruited through the Swedish Colorectal Cancer Low-Risk Study. All 775 cases of breast cancer used for evaluating the recessive variant were collected from the Department of Clinical Genetics, Karolinska University Hospital. The 328 cases of sporadic breast cancer samples used in validation of non-synonymous variants were collected from a population-based cohort from Södersjukhuset, Stockholm. Genomic DNA was extracted from peripheral blood samples. Samples were genotyped using TaqMan SNP genotyping assay (Thermo Fisher Scientific, Waltham, Massachusetts.

### Finnish validation cohorts

Rs16897117 was genotyped in two breast cancer cohorts from the Helsinki region, one including 1721 unselected cases and 755 additional familial cases^18,31–33^ and another consisting of 993 unselected cases^34^, as well as in a cohort of 666 breast cancer patients from the Tampere region, described in detail previously^31,33^ (Table 1). *CHEK2*:c.1100delC genotype data were readily available from one of the Helsinki cohorts^35^, the other two Finnish cohorts were genotyped for c.1100delC with a TaqMan assay.

### BCAC data

The BCAC data used for final validation of the rs16897117 was retrieved from the OncoArray project, described previously^5^. We included in the analysis the independent studies participating in the consortium, if there was sufficient data on reliably imputed c.1100delC available (at least 10 carrier cases and 10 healthy carrier controls per study). Only the study subjects with European ethnic background were included, and the Swedish and Finnish cohorts included in the discovery analyses were excluded. The selection yielded 13,767 breast cancer cases and 21,456 controls (Table 2).

### Statistical analysis

Odds ratios, 95% confidence intervals and p-values were calculated to test the association with allele frequency using the DeFinetti programme provided as an online source^36^. The validation analyses were performed using R environment for statistical computing version 3.6.1 (R Core Team (2019)^37^. For the case-only analysis of the Swedish and Finnish cohorts, a stratified Mantel–Haenszel odds ratio was estimated with R library *epiDisplay*^38^*.* The BCAC data analysis was performed with logistic regression. The interaction between c.1100delC and rs16897117 was assessed with likelihood-ratio test.

### Ethics declaration

This study was approved by the Ethics Committee of Karolinska Institutet/Karolinska University Hospital. All individual studies, from which data was used, were approved by the appropriate medical ethical committees and/or institutional review boards. All methods were performed in accordance with the relevant guidelines and regulations. All study participants provided informed consent.
